# Multivariate Assessment of Procedures for Molecularly Imprinted Polymer Synthesis for Pesticides Determination in Environmental and Agricultural Samples

**DOI:** 10.3390/ma14227078

**Published:** 2021-11-22

**Authors:** Mariusz Marć, Marta Bystrzanowska, Katarzyna Pokajewicz, Marek Tobiszewski

**Affiliations:** 1 Department of Analytical Chemistry, Faculty of Chemistry, Gdansk University of Technology (GUT), 80-233 Gdansk, Poland; marbystr@student.pg.edu.pl; 2 Department of Analytical Chemistry, Chemical Faculty, Opole University, 45-040 Opole, Poland; katarzyna.pokajewicz@uni.opole.pl; 3 Department of Analytical Chemistry, Faculty of Chemistry and EcoTech Center, Gdansk University of Technology (GUT), 80-233 Gdansk, Poland; martobis@pg.edu.pl

**Keywords:** molecularly imprinted polymers, pesticides, environmental samples, agricultural samples, multi-criteria decision analysis

## Abstract

In the case of quantitative and qualitative analysis of pesticides in environmental and food samples, it is required to perform a sample pre-treatment process. It allows to minimalize the impact of interferences on the final results, as well as increase the recovery rate. Nowadays, apart from routinely employed sample preparation techniques such as solid-phase extraction (SPE) or solid-phase microextraction (SPME), the application of molecularly imprinted polymers (MIPs) is gaining greater popularity. It is mainly related to their physicochemical properties, sorption capacity and selectivity, thermo-mechanical resistance, as well as a wide range of polymerization techniques allowing to obtain the desired type of sorption materials, adequate to a specific type of pesticide. This paper targets to summarize the most popular and innovative strategies since 2010, associated with the MIPs synthesis and analytical procedures for pesticides determination in environmental and food samples. Application of multi-criteria decision analysis (MCDA) allows for visualization of the most beneficial analytical procedures in case of changing the priority of each step of analysis (MIPs synthesis, sample preparation process—pesticides extraction, chromatographic analysis) bearing in mind metrological and environmental issues.

## 1. Introduction

Over the last years, the incorporation of materials defined as molecularly imprinted polymers (MIPs) to extraction, microextraction as well as solventless extraction techniques has been a field of research in which the scientific activity is still growing. Nowadays, molecularly imprinting technologies (MITs) are constantly improved and developed to create new types of selective materials that might be successfully introduced in analytical processes associated with environmental, food and biological samples analysis. In laboratory practice, MIPs might be considered as an important element of chromatographic columns (especially for liquid chromatography) and electrochemical sensors. Nevertheless, the leading field of research that involves the application of various types of MIPs is still the sampling and sample preparation process. This phenomenon is mainly caused by the wide spectrum of beneficial properties of MIP materials—high extraction recovery, high stability, chemical and mechanical resistance, reusability as well as relatively simple preparation under laboratory conditions [1,2,3]. According to Köse et al., 2021, the MIP developing process (including bulk, precipitation, suspension, emulsion, etc. polymerization techniques) is associated with the principle of creating template-specific polymeric cavities fitted to the target molecules in the presence of selected chemicals through non-covalent (hydrogen bond, ionic, and hydrophobic) or covalent interactions [4,5]. The presence of specific cavities that are created by the polymeric skeleton causes that developed polymeric materials are characterized by high selectivity to defined specific chemical compound or a specific group of chemical compounds. The MIPs’ abilities for selective recognition/sorption of target chemical compounds from the samples that are characterized by complex matrix composition gives a possibility to reduce the matrix effect on the final results, improve the precision, accuracy and the values of method detection limits (MDL). Additionally, the application of mentioned selective polymeric materials in a sample preparation/analytes extraction stage is directly linked to the philosophy of green analytical chemistry (GAC) mainly by decreasing the hazardous liquid and solid wastes, minimalizing the sample and organic solvents consumption and reducing the number of steps of analytical procedure [6,7].

**Figure 1:**
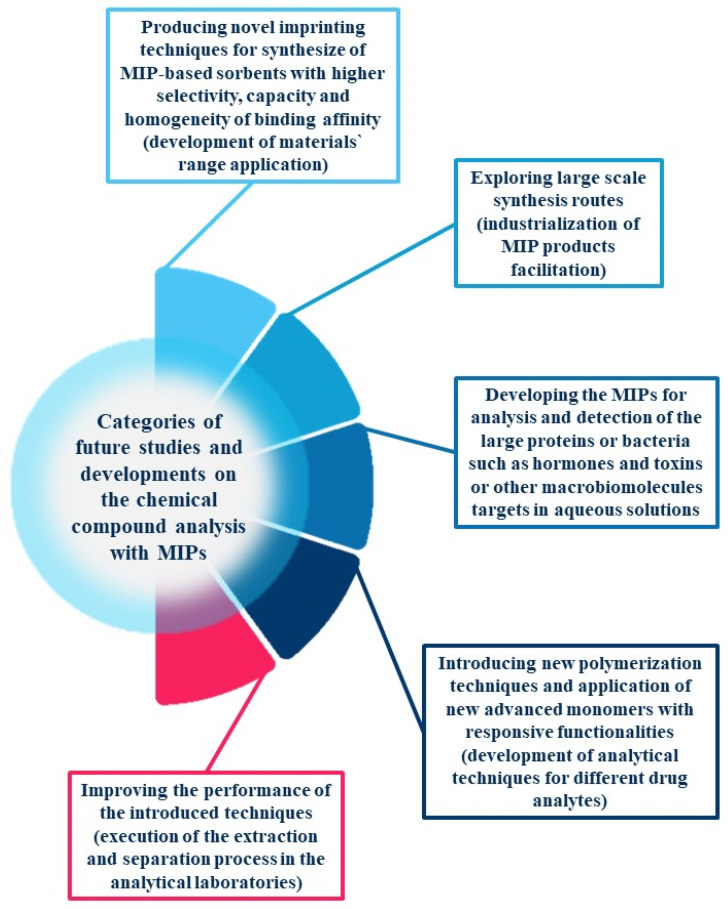
Categories of future research and developments on the chemical compounds analysis with MIPs [5].

Unfortunately, MIP might be considered as a very convenient and “green” sorbent due to its physicochemical properties and high selectivity only as a ready-to-use solid material—at the stage of its application during the defined analytical protocol. Considering the whole process that is necessary to obtain the desired form, shape and quality of MIP sorbent, the “green” character of such material might be the subject for discussion. Sarafraz-Yazdi and N. Razavi (2015) mentioned that finding a suitable sorption material for extraction/isolation of an analyte is challenging due to the fact that several parameters should be considered such as stability, suitable selectivity, high sensitivity, fast response. Preparing a sorbent with all of the mentioned features is unachievable, and it is strongly advised to sacrifice some features in favor of others [8]. Most of the reported analytical protocols for the selective isolation/extraction and determination of xenobiotics in environmental, food and biological samples using conventional MI-SPE technique requires to use of a large volume of organic solvents and the imprinted polymer preparation process is based on classical bulk polymerization—heterogeneous distribution of particles both in shape and size. It shows that even in the field of potentially “green” materials there is a growing interest in alternative routes of imprinted polymers preparation [9]. Ansari and Karimi (2017) report that the future research associated with the MIPs development process (regardless of the area of their potential application) should consider several principles, that were shown schematically in the Figure 1 [5].

For this reason, an attempt should be made to consider MIPs in a heliocentric manner and take into account not only the aspect related to their main area of application (analytes extraction/isolation techniques), but also the process of their preparation (synthesis) and the type and quality of the system used at the final determination stage should be assessed. 

In modern agriculture to efficiently protect crops from negative impact of pathogens or weeds, a large number of different pesticide agents are used, among them insecticides, fungicides, herbicides, rodenticides. They are characterized by a different source of origin, chemical structures, formulation, toxicity, mode of entry and action. [10,11]. Furthermore, they can contaminate the surrounding environment or even bioaccumulate. Even when applied sustainably, there is always a risk of unplanned, negative impact on human health and other life forms. Thus, the levels of pesticides and their metabolites must be continuously monitored in food and the environment [12,13].

The procedures of pesticides analysis in food and environmental samples are often time and labor-consuming and use a large volume of organic solvents and plastic consumables. This is mostly due to the complex sample preparation step. To overcome this, many solutions are being examined, starting from well-established techniques such as QuEChERS, SPE and SPME, ending on sophisticated microextraction techniques and sensors. Majority of these techniques utilize some sort of sorbent. One of the most interesting trends, in the development of analytical methods for pesticides, is the use of various combinations of MIP materials (sorbents). Their advantage is high selectivity and what is very important—they are low-cost materials that can be easily prepared in contrast to biological origin receptors such as enzymes, aptamers, anti- or nanobodies. Therefore, they have been widely studied and applied in SPE (MI-SPE), (magnetic) dispersive SPE (mag-MIP), SPME sample pretreatment applications and sensors (QDs or CDs) for different pesticides from various chemical classes. These applications have been already reviewed by different authors with a focus on pesticides specifically [12,14,15,16,17,18] or on a specific MIP-using technique, but including some pesticides applications [8,19,20]. Introducing slight modifications (performed with rather routine laboratory equipment) into the MIP preparation process, new type of selective polymeric sorption materials might be successfully combined with a wide spectrum of sample preparation extraction and microextraction techniques without losses in selectivity, stability and reusability [9].

The goal of the performed and described study is finding interior patterns in the dataset characterizing entire analytical protocols (MIPs synthesis, sample preparation process—pesticides extraction, final determination) for representatives of pesticide residues determination in environmental and agricultural samples and selection of the most suitable one from among: 11 protocols which use MI-SPE technique; 12 procedures which employ mag-MIP materials; 9 methods which apply nanomaterial-based MIPs (QDs or CDs). The application of multi-criteria decision analysis (MCDA) combined with sensitivity analysis allows for sufficient visualization of the most beneficial analytical procedures considering GAC principals. The use of Cluster Analysis (CA) allows to select the groups of variables or reduce the number of variables to multi-criteria decision analysis. At this juncture, to overcome the issue of subjectivity of weights assignment the ternary graph is applied to show the ranking effects for the entire range of weights assigned to defined criteria.

## 2. Materials and Methods

### 2.1. Dataset Collection, Alternatives and Criteria

The input data for statistical analysis are parameters characterizing different approaches to molecularly imprinted polymers synthesis and analytical procedures for pesticides determination in environmental and agricultural samples. Considered criteria relevant from metrological and environmental points of view are presented in Table 1. Intuitively, the criteria are divided into three groups related to MIPs synthesis (MIP preparation), sample preparation process—pesticides extraction (MIP application) and chromatographic analysis (final determination). In the following studies, analytical protocols related to use of MI-SPE, magnetic MIP and QD or CD techniques described in the scientific literature are evaluated (for details see Table 2, Table 3 and Table 4). Only fully characterized analytical procedures are taken into analysis (according to selected parameters).

**Figure 2:**
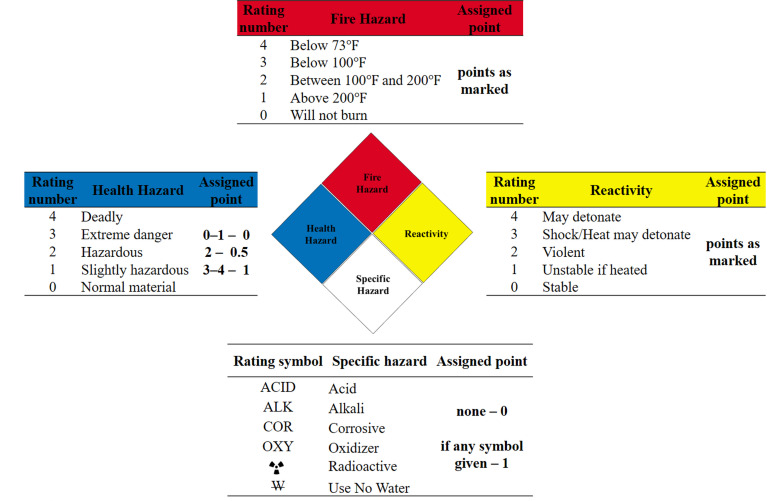
Points assessment guideline with use of Hazard NFPA 704 System.

Some of data require transformations from descriptions to numerical values, which is needed for further use of MCDA and chemometric techniques. To identify the hazards associated with material the modification of the system proposed by The National Fire Protection Association (NFPA 704) was applied [21]. The system uses a color-coded diamond with four quadrants in which numbers are used in the upper three quadrants to signal the degree of: health hazard (blue), flammability hazard (red), and reactivity hazard (yellow). The bottom white quadrant is used to indicate special hazards described by the rating symbol. Using the above classification, the relevant quadrants and symbols have been given points, the sum of which is the result of the assessment of the hazard potential of a given chemical substance. This procedure of chemicals greenness assessment may look oversimplified, but it has the advantage of bringing many aspects into a single score. Moreover, the pictograms are readily available for the great number of compounds. The method of assigning points for group of factors is presented in Figure 2.

In case of the majority of descriptive criteria, points are allocated in the zero-one system. The details for points assessment are summarized in Table 1.

### 2.2. Cluster Analysis

Finding some similarities and differences between the elements under assessment is possible via chemometric method called Cluster Analysis (CA) [53]. This multivariate statistical tool allows for splitting the variables or objects into reasonably homogeneous groups according to the similarity or dissimilarity of elements. In case of clustering without supervision, the unsupervised algorithm finds internal patterns in the dataset with no a priori information or assumptions on the dataset. More details about the algorithm may be found elsewhere [54]. The nomenclature of elements in chemometric methods includes variables and objects represented by parameters and possible options, respectively. In CA, similarity (dissimilarity) of elements may be determined by definition of the distance in multivariable space. The grouping of variables and objects is performed with Euclidean distance measure and Ward cluster formation method. After standardization of initial dataset, calculations for CA are performed with Statistica12 software (StatSoft). 

### 2.3. Technique for Order of Preference by Similarity to Ideal Solution

In this study, a TOPSIS algorithm (Technique for Order of Preference by Similarity to Ideal Solution) developed by Hwang and Yoon, as one of MCDA technique, is used [55,56]. TOPSIS is selected as it is very easy in use, interpretation of the result and performs well with a great number of criteria. The aim of MCDA application is finding the most favorable option (an analytical procedure for pesticides determination in environmental and food samples) among all available ones, what is possible due to obtained ranking results. The assessment procedure for MCDA can be performed in few steps involving, stating of the aim, criteria and alternatives identification, weighting of criteria and then algorithm application with results interpretation [57]. MCDA techniques use a specific nomenclature: criteria—are parameters that describe available options (e.g., type or amount of chemical reagents, time of analysis) and alternatives—are available options taken into assessment (analytical procedures). It is very important that, all the factors must be in a form of numerical values, or they must be easily transformable into them. The weighting of criteria is step where appropriate weight values are assigned to each criterion, to indicate their relative importance. The basis of the TOPSIS algorithm involve the input data as a matrix consisting of n alternatives and described by m criteria and its mechanisms can be described in several steps:

1. Construction of normalized decision matrix

(1) $$r_{ij}=x_{ij}÷\sqrt{\sum x_{ij}^{2}},\;i=1,2,\ldots ,m\wedge j=1,2,\ldots ,n\;$$

where *x_ij_* and *r_ij_* are original and normalized scores in decision matrix, respectively.

2. Construction of the weighted normalized decision matrix

(2) $$v_{ij}=r_{ij}\times w_{j},\;i=1,2,\ldots ,m\wedge j=1,2,\ldots ,n\;$$

where *w_j_* is the weight of the criterion j and $\overset{n}{\underset{j=1}{\sum }}w_{j}=1$

3. Determination of positive ideal (A^*^) and negative ideal (*A*^−^) solutions

(3) $$A^{*}=\left\{\left({max}_{i}v_{ij}|j\in C_{b}\right),\left({min}_{i}v_{ij}|j\in C_{c}\right)\right\}=\left\{v_{i}^{*}|j=1,\;2,\;\ldots ,\;m\right\}$$

(4) $$A^{-}=\left\{\left({min}_{i}v_{ij}|j\in C_{b}\right),\left({max}_{i}v_{ij}|j\in C_{c}\right)\right\}=\left\{v_{j}^{*}|j=1,\;2,\;\ldots ,\;m\right\}$$

4. Calculation of the separation measures for each alternative

(5) $$S_{i}^{*}=\sqrt{\overset{m}{\underset{j=1}{\sum }}{\left(v_{ij}-v_{j}^{*}\right)}^{2}}j=1,\;2,\;\ldots ,\;m\;$$

(6) $$S_{i}^{-}=\sqrt{\overset{m}{\underset{j=1}{\sum }}{\left(v_{ij}-v_{j}^{-}\right)}^{2}}j=1,\;2,\;\ldots ,\;m\;$$

5. Calculation of the relative closeness to the ideal solution

(7) $$C_{i}^{*}=\frac{S_{i}^{-}}{S_{i}^{*}+S_{i}^{-}},\;i=1,\;2,\;\ldots ,\;m\;0<C_{i}^{*}<1\;$$

6. Arrangement of scenarios in order of closest to ideal to furthest from ideal—creation of the ranking

As presented above, in case of TOPSIS algorithm, finding the most favorable option is based on selection of the alternative that simultaneously has the shortest distance to the positive ideal solution and at the same time the farthest distance to the negative ideal solution. Therefore, the alternative with $C_{i}^{*}$ closest to 1 is the best preference among the possible options. The TOPSIS calculations are performed in Microsoft Excel 2016 program (Microsoft, Redmond, WA, USA).

## 3. Results

Each of sets of procedures was subjected to grouping with CA to find the similarities and differences between the procedures under assessment. This is helpful in finding greener solutions or rejecting the procedures that are definitely non-green. Procedures were also assessed with TOPSIS analyses, with the application of different weights to find the greenest solutions. The weights are presented with ternary plots, the assessment criteria were grouped according to the procedural stage as shown in Table 1. Each of corners of ternary plot gives the information on the winners according to rankings with criteria relevant to respective stage of procedure only. The remaining surface of the ternary plot presents the winners according to rankings with mixed weights for sets of criteria.

### 3.1. The Greenness Assessment of MI-SPE Technique for Pesticide Determination

**Figure 3:**
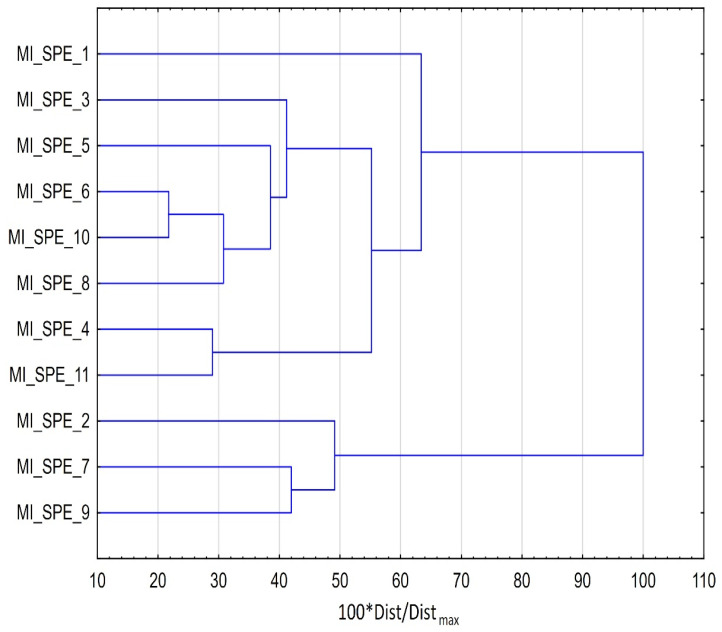
Grouping of procedures based on MI-SPE, obtained with cluster analysis.

The clustering results, presented in Figure 3 clearly indicate that two groups are formed, the first one contains 8 procedures, the second one only three. The discriminators between groups are gas chromatographic analysis applied in the latter three procedures and considerably higher amounts of functional monomers, cross-linking agents, and porogens and considerably large amount of MIP applied (for the discriminators it is needed to see raw dataset gathered based on the information published in papers listed in Table 2). In the larger cluster it is hard to find and discuss the further mechanisms of clustering.

MI-SPE sorbents are usually produced using a non-covalent imprinting approach and can be synthesized using bulk, precipitation, suspension, emulsion and multistep swelling polymerization methods. The most common is bulk polymerization, followed by precipitation polymerization [58,59]. In bulk polymerization, the reaction mixture forms a polymer block that must be mechanically ground and sieved through a sieve of an appropriate size (usually 25–50 µm) [16,60,61]. In precipitation polymerization, the reagents are very similar except for the solvent (porogen agent), which amount is much bigger—usually 2–10 times. Proper process conditions and reagents composition allow obtaining micro- and nanoscale regular particles with higher efficiency. The big advantage of this technique is no necessity to grind and sieve the polymer. On the other hand, a big disadvantage is the usage of a large volume of solvent [60,61]. Both methods, especially bulk polymerization, are relatively easy and cheap techniques that can be performed in a laboratory equipped with basic chemical instruments and tools. The resulting sorbent can be straightforwardly utilized in different SPE formats. This is one of the reasons why MI-SPE gained popularity and is used in research/analytical laboratories worldwide. Obviously, the main reason for the growing attention of molecularly imprinted sorbents is their unique combination of selectivity with high thermal, chemical and mechanical stability [14]. 

From the analytical practice point of view the application of MI-SPE is often referred as a green analytical technique, due to reduce the amount of used resources such as energy and reagents (especially toxic ones) and minimize the generation of produced waste [62,63]. Regarding MI-SPE, sorbent reusability is usually highlighted as a green chemistry feature as it limits the amount of solid waste [64]. Furthermore, the selectivity of MI-SPE and its efficiency of isolation and enrichment of analytes meet assumptions of GAC. On the other hand, considering the preparation process of appropriate MIP sorbent, the reagents used in the synthesis of MIPs are known to be hazardous and they are used in significant volumes, not only during final synthesis, but also during process optimization or in case of necessity to repeat the reaction if a MIP product does not meet desired specification. Such repeated experiments result in a large waste generation which ends up in contradiction with the green chemistry ideals [65]. Therefore the “greenness” of MI-SPE analytical methods is a matter of discussion. To evaluate it, two main issues have to be assessed: (i) factors associated with the imprinted sorbent production and (ii) factors concerning the application of developed MIP sorbent, in a given SPE configuration and analytical procedure [63]. It is a complex issue and the ratings can differ and depend on the synthesis method, reactants, MI-SPE configuration and SPE technique. 

In preparation of MI-SPE sorbents, as seen in Table 2, the most common choice for monomer reagent is methacrylic acid (MAA), ethylene glycol dimethacrylate (EGDMA) as cross-liner, azodiisobutyronitrile (AIBN) as for an initiator and acetonitrile (ACN), toluene, dichloromethane (DCM) or chloroform as porogen agent. The template/monomer/cross-linker molar ratio is most frequently 1:4:20. This ratio is probable to provide optimum imprinting efficiency [66] and in the most cases the mentioned value of the molecular ratio is confirmed by the results of computer molecular modelling. The proportion of reagents is very important in non-covalent imprinting as it requires the presence of template-monomer complexes. To shift the equilibrium in right—towards the formation of the template-monomer complex, it is necessary to use an excess of monomers in relation to the template, but that also carries the risk of formation of non-specific binding sites [67]. In order to obtain the desired properties of the MIP adsorbent, the preparation process of the polymer should be optimized. This is not straightforward as there are many parameters to optimize [68]. Consequently, the development of MIP sorbents requires a lot of tedious laboratory work that consumes time and reagents. Computer-assisted molecular modelling can support the process of synthesis optimization. It can help to reduce the number of chemical reactions that may fail, decrease consumed reagents, energy and produced waste [69].

As stated above, the synthesis and MIP sorbent preparation is quite demanding regarding consumed reagents and energy. Before synthesis reactants must be subject to purification to remove polymerization inhibitors. The reaction mixture is dissolved in toxic solvents and the polymerization is typically conducted for many hours up to one day in elevated temperatures [64]. The biggest consumption of solvents for both polymerization methods is related to the template removal. It is usually performed in a Soxhlet device and takes many hours, sometimes is repeated several times and consumes a large volume of solvents (usually acidified MeOH) [59].

Not only the MIP preparation process consumes a lot of solvents and reagents, but also the application stage of MI-SPE as a sample pre-treatment technique (mainly working in the off-line mode) [58]. A small amount of imprinted sorbent is packed in a cartridge and must be: (i) conditioned, (ii) sample loaded, (iii) washed and (iv) eluted. All these activities are quite laborious and consume a further amount of time, toxic organic solvents and electricity. Typically, the total volume of organic solvents used for one MI-SPE extraction (all four steps) equals several up to 20 mL. At the end, the eluate is often further processed—dried with nitrogen and reconstituted with an appropriate organic solvent or its mixture with water. As described above, despite many advantages of MI-SPE the entire process is not very straightforward and very green. It consumes a lot of resources such as time, lab work, reagents (including toxic organic solvents), some consumables and energy. Additionally, it should be highlight that after MI-SPE clean-up procedure big amounts of organic solvents such as MeOH or ACN are used during HPLC analysis.

The example of MI-SPE procedure with sorbent obtained by precipitation polymerization method was described by Zuo et al. (2015). The authors prepared MIP for insecticide—malathion and showed its usability as a sample pretreatment technique in different types of matrices such as tap water, soil and cabbage. MIPs were prepared using precipitation polymerization and malathion as a template. Firstly, they allowed for the formation of template-monomer complexes by mixing malathion (0.033 g) and MAA (0.516 g) with 200 mL of ACN-chloroform by ultrasonic bath for 5 min and kept in the refrigerator overnight. One day later EGDMA (5.94 g) and AIBN (90 mg) were added, the solution was mixed and purged with a stream of nitrogen and closed tightly. The polymerization was conducted at 70 °C for 10 h. The obtained imprinted polymer was washed with three 20 mL aliquots of ACN, then with three 20 mL aliquots of MeOH-acetic acid, once with 20 mL of water and with three 20 mL aliquots of MeOH. Afterwards, MIP was dried in the oven (60 °C) and then subjected to 24-h long extraction using MeOH-acetic acid to remove the malathion template. Furthermore, MIP was washed with water and MeOH to remove the acetic acid residues and dried at 60 °C. The SPE procedure with the obtained imprinted material was following conditioning—4 mL MeOH and 4 mL water; loading of sample—20 mL tap water or samples extracts (water-MeOH); washing—8 mL MeOH-water followed by vacuum sorbent drying; finally, the SPE tube was eluted with 8 mL MeOH-acetic acid. Elute was dried under nitrogen (40 °C) and reconstituted in ethyl acetate for GC analysis. In addition, the authors investigated the reusability of MIP by repeating the cycle adsorption–elution four times. They found out that recovery of the fourth time was still more than 95%, indicating that the sorbent can be reused at least 4 times. Such recyclability of MIP material helps to limit solid waste when compared to routine single-use SPE tubes, but still, some organic solvents have to be used for regeneration of cartridges [22].

Another exemplary procedure, this time using bulk polymerization might be the one described by Martins et al. (2015). The authors used MI-SPE for sample pretreatment in deltamethrin determination in olive oil. In this case, acrylamide (36 mg), which turned out to be better than commonly used MAA was employed. Next, the EGDMA (0.48 mL), deltamethrin (63 mg) and DCM (2.4 mL) were mixed. Lastly AIBN (40 mg) was added and the whole flask was sonicated under a nitrogen atmosphere for 10 min. Polymerization was performed at 60 °C for 24 h. Template wash was carried out with MeOH-acetic acid solution until no template was present. The second purification was carried out for 24 h for removals of acidic residuals. The washed and dried sorbent was further utilized in the SPE procedure (50 mg per analysis). The SPE procedure was following conditioning—5 mL MeOH and 5 mL heptane; loading of sample—1 g of spiked olive oil diluted with 5 mL of heptane; washing—2 mL of heptane followed by 1 mL of heptane containing 10% of DCM; finally, the analyte was eluted with 1 mL of MeOH. Elute was dried and reconstituted in acetonitrile for HPLC-DAD analysis [23].

**Figure 4:**
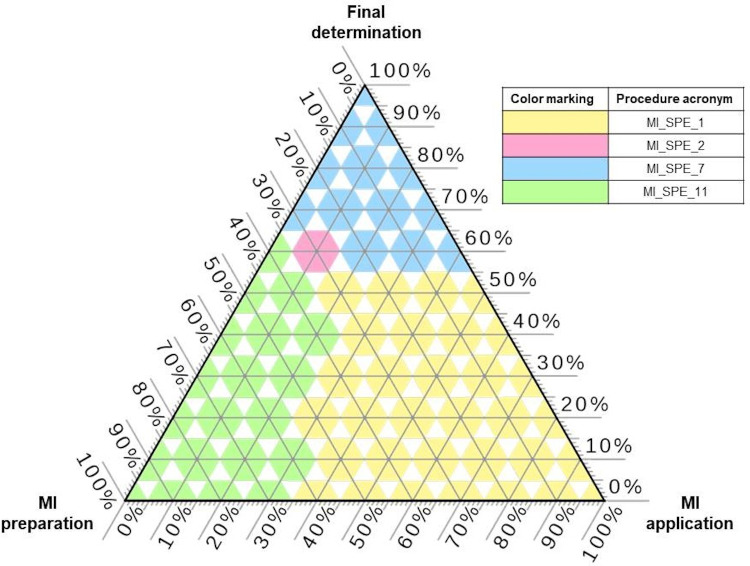
The winning procedures based on MI-SPE according to TOPSIS rankings for different weights combinations.

Considering only the analytical application of MI-SPE in mentioned examples, prepared MIPs seem to meet the assumptions of GAC. The organic solvent consumption is less than 20 mL per sample. Nevertheless, detailed direct comparison of solvent and reagents consumption for all of the listed in Table 2 MI-SPE application solutions is very difficult due to multiple steps of a whole analytical procedure as well as a high number of influencing factors on the final application of MIP. Therefore, some general assumptions and simplifications must be carried out. For this reason, detailed data was collected from the references listed in Table 2 and a special type of database was created to assess which stage of employment of MI-SPE (MIP synthesis, MIP analytical application, final determination of desired analytes) has the most significant effect on increasing/decreasing the green character of the entire analytical procedure. Figure 4 shows the winning procedures for different weights combinations. For predominant weights of MIP preparation greenness, the procedure MI_SPE_11 is the most beneficial, as it requires low amounts of all reagents and the functional monomer is itaconic acid that is assessed as greener than predominantly used in other procedures methacrylic acid. When the stage of MIP application is considered the most beneficial procedure is MI_SPE_1 that involves only 30 mg of MIP and only 1 mL of elution solvent (some others need 5 or 8 mL) and low volumes of additional solvents. The winning procedure for final determination dominant weights is MI_SPE_7 that applies rather non-problematic GC-FPD detection system that allows to reach acceptable metrological parameters values. For very specific weights (60% for final determination, 30% to MIP preparation and 10% to sample preparation) the winning procedure is MI_SPE_2 but this finding is of lesser importance. This ternary plot indicates which MIP preparation, sample preparation and final determination steps should be selected in future development of analytical procedures.

### 3.2. The Greenness Assessment of Magnetic MIP Technique for Pesticide Selective Recognition from Food and Environmental Samples

**Figure 5:**
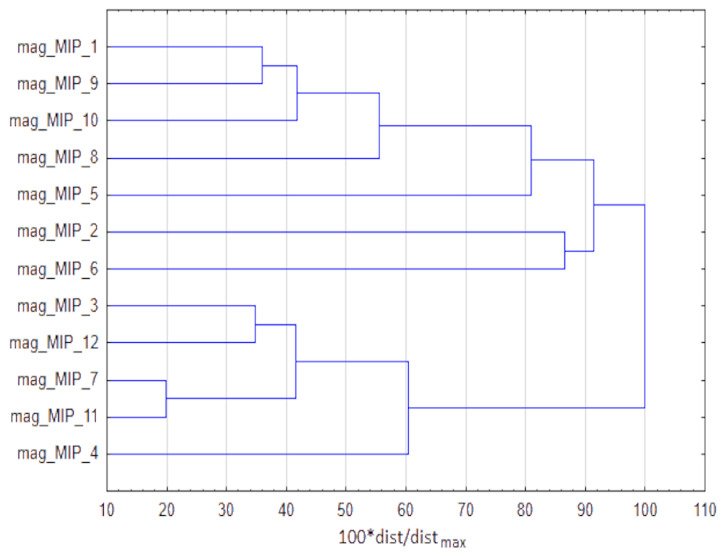
Grouping of procedures based on mag-MIPs, obtained with cluster analysis.

Figure 5 presents the clustering of procedures that are based on mag-MIPs application. The clustering is obtained with CA as described before. Two clusters are formed, the first one with 7 procedures, the second one with 5 of them. The difference between them is not that clear but it results from the fact that procedures in the second cluster generally need lower amounts of washing solvents, functional monomers and porogenic agents (but the larger cluster contains also fewer material-consuming procedures; for the discriminators it is needed to see raw dataset gathered based on the information published in papers listed in Table 3).

Mag-MIPs are one of the most specific representatives of products resulting from the application of the surface coating/imprinting technique. In general, the selected type of core (materials include iron, cobalt, nickel and their oxidizing material) characterized by magnetic properties is covered by the thin-film of sorption material (polymer) that possesses specific binding sites. Currently, in analytical practice, the most popular magnetic cores are based on iron oxide Fe_3_O_4_ (due to low toxicity and low costs in relation to other materials). The mag-MIPs developing process is mainly based on several stages such as: (i) preparation or synthesis of magnetic particles/nanoparticles; (ii) surface modification and functionalization; (iii) the attachment of template molecules or its structural analogue; (iv) polymerization process and (v) removing the template or dummy template molecules [70]. After obtaining the magnetic nanoparticles and after completing the polymer sorbent covering process, achieved materials might be successfully applied in a sample preparation stage, due to the fact that they can be easily separated from the investigated sample with the external magnet. Moreover, appropriately prepared mag-MIPs are characterized by large imprinted and uniform surface, easier rebinding progress of target molecules with the recognition sites, higher adsorption capacity and lower leakage of template (in comparison to traditional bulky polymerization), high selectivity as well as high sensitivity. Taking into account all of the mentioned advantages, form their users’ point of view, ready to use mag-MIPs are very promising and powerful tools in sample preparation, analytes separation and extracts purification fields [14,71,72]. 

From the application point of view, mag-MIPs might be considered as a convenient solution for rapid analytes adsorption and their direct separation from the liquid samples characterized by complex matrix composition. Furthermore, the ready to use mag-MIPs follows the canons of the philosophy of GAC by decreasing the number of steps in the analytical procedure. Mag-MIPs application procedure in analytical practice mainly consists of four steps: (i) incubation of dispersed mag-MIP with investigated liquid sample by defined time period; (ii) recovery of mag-MIP particles from the bulk solution using external magnet; (iii) elution/desorption of analytes collected by employed mag-MIP particles using a proper solvent; (iv) analytes separation, identification and final determination [73]. The introduction to the analytical protocol magnetic materials supported with a properly selected polymer sorbent reduces the volume of the sample and consumed organic solvents (without losing the efficiency of the extraction process). Additionally, small amounts of solid sorbents (several dozen grams which might be used several times) are used during a single analysis; as a consequence, small amounts of solid waste are generated. 

Nevertheless, the “green nature” of this type of materials should be considered holistically, taking into account the process of obtaining cores with magnetic properties along with the stage of applying/covering the surface of magnetic core with a thin layer of MIP material. The development process of appropriate magnetic sorption materials often needs a complicated and precise multi-step modification, is very time-consuming as well as requires a relatively large number of solvents and reagents. The most critical points concern obtaining the appropriate diameter of Fe_3_O_4_ particles and the proper conduct of the process of covering the magnetic cores with a thin layer of a suitable carrier and then attaching the appropriate MIP material. Another challenging point during the MIP synthesis and application of mag-MIPs is a highly probable the phenomenon of the leakage of template molecules—very important issue especially during the determination of trace analytes [36]. In laboratory practice, Fe_3_O_4_ nanoparticles are mainly prepared employing solvothermal and co-precipitation techniques. The most popular and widely applied solution is the co-precipitation technique due to its easy operation, simple apparatus and mild synthesis conditions. The second technique—solvothermal, is less popular than the co-precipitation and is prepared in water phase under high pressure and temperature conditions. Iron oxide nanoparticles prepared using this solvothermal technique are characterized by integrity structure, less aggregation between particles as well as uniform particle dimensions [72,74]. In order to show the complexity of the preparation of polymer sorbents on a magnetic carrier and the type and potential amount of used solvents and reagents, two different examples of the methodical approach are presented below.

An interesting, preliminary solution associated with the application of magnetic solid sorbent supported by imprinted polymer was described by Díaz-Alvarez et al., (2019). In this work, the combination of SPE technique, magnetic particles and MIP was reported as a self-made novel imprinted SBSE element for selective isolation of representatives of fungicides (thiabendazole and carbendazim) from citrus samples. Considering only the first stage of the entire laboratory practice, the preparation of mentioned novel SBSE tool was performed in two general steps—modification of magnetic nanoparticles and preparation of magnetic imprinted stir-bars. At the beginning, the surface of magnetic nanoparticles (200 mg) was modified with the use of oleic acid (water solution containing 0.06% of oleic acid (OA)) at temperature 80 °C for 25 min, and at the end washed several times by water and MeOH. After this, applying the conventional sol-gel technique, the encapsulation of prepared magnetic nanoparticles inside a silica network was implemented. Freshly obtained nanoparticles of Fe_3_O_4_@OA were dissolved in 54 mL of 2-propanol water solution and sonicated to disperse nanoparticles. Next, 5 mL of solution of ammonia in water (25%) and 2 mL of tetraethyl orthosilicate (TEOS) were introduced sequentially. The mixture was kept in continuous stirring at room temperature for several hours and then rinsed few times by water, MeOH and dried. At the end of this stage, the defined amount of freshly prepared Fe_3_O_4_@OA@SiO_2_ magnetic nanoparticles were dispersed (ultrasounds support) in 50 mL of MeOH and then 3 mL of 3-methacryloyloxypropyltrimethoxysilane (MPS) was introduced drop by drop (reaction time—24 h) to obtain methacrylate-modified surface of magnetic nanoparticles. As for the second stage of the preparation of SBSE tool (preparation of magnetic imprinted stir-bars employing modified bulky polymerization technique), the defined amount of methacrylate-modified Fe_3_O_4_@OA@SiO_2_ magnetic nanoparticles was introduced to the 0.5 mL of glass vial insert as a reaction vessel. Then, an aliquot of 0.5 mL of the optimum polymerization mixture (prepared in a 3.46 mL of a toluene-ACN solvent—porogen agent), containing 58.4 mg of thiabendazole (template molecule), 157.4 mL of MAA (functional monomer), 1.0 mL of EDGMA (cross-linking agent) and 33.5 mg of AIBN (reaction initiator). After agitation in a vortex and incubation (temp. 60 °C) using a low-profile roller by 4 h, the obtained monolith was removed from the reaction vessel by crushing the insert. At the end of the whole SBSE MIP preparation procedure, achieved magnetic solid material was washed several times by MeOH-acetic acid mixture to remove the template [32]. Similar approaches in the field of developing the novel SBSE imprinted solutions for the determination of pesticides in environmental samples were described elsewhere [75,76]. 

Different, more complicated and requiring more precision solution in the field of obtaining appropriate functional magnetic nanomaterials for pesticides determination in environmental and food samples are magnetic core-shell mesoporous MIPs—specific type of magnetic dispersive solid-phase extraction (MDSPE). In general, the preparation of mentioned mag-MIPs consists of two main steps—preparation and modification of magnetic microspheres (Fe_3_O_4_) and preparation of magnetic core-shell mesoporous MIP. He et al., (2019) describe the evaluation of magnetic core-shell mesoporous MIPs for selective collection of defined residues of pesticides in food samples. The authors prepared magnetic nanoparticles of Fe_3_O_4_ based on the self-modified chemical co-precipitation technique. In 10 mL of ultrapure water, defined mass of FeCl_3_·6H_2_O and FeCl_2_·4H_2_O were dispersed. After this, the prepared solutions were moved to appropriate glass flask equipped with a stirrer and additional 80 mL of ultrapure water was introduced (stirring time—10 min). Next, the 10 mL of ammonium hydroxide (25% solution) was added and the mixture was stirred for 30 min at elevated temperature (80 °C). Afterwards, defined mass of citrate tribasic dehydrate was introduced into the heated mixture and stirred for additional 30 min. Obtained black Fe_3_O_4_ nanoparticles were washed several times by ethanol and water. The preparation of magnetic core-shell mesoporous MIP was performed according to the following procedure. A defined mass of freshly prepared Fe_3_O_4_ was placed in a glass vessel and dissolved in 180 of EtOH:water solution (2:1), supported by ultrasounds for 30 min. Afterward, 1.5 mL of 3-aminopropyltriethoxysilane (APTES) was introduced and whole mixture was stirred at slightly elevated temperature (40 °C) for 30 min (creation of Fe_3_O_4_-NH_2_ connections). Next, in 10 mL of ACN, the 0.294 triadimefon (template molecule) together with 50.89 μL of MAA (functional monomer) were preincubated for 30 min. Modified nanoparticles of Fe_3_O_4_-NH_2_ were dispersed in 10 mL of ACN (porogen agent) and 319 μL of trimethylolpropane trimethacrylate (cross-linking agent) and mixed together with 40 mg of AIBN (reaction initiator). The tightly sealed glass flask was kept in elevated temperature (60 °C) for 24 h. After the polymerization process, potential reagents residues as well as template molecule were washed out and prepared mag-MIP sorbent was dried under vacuum conditions [33]. 

As can be seen, the discussed exemplary solutions in the field of mag-MIP clearly indicate the relatively complex and time-consuming process of obtaining magnetic cores and covering them with a suitable layer of selective polymeric material. Consequently, this leads to the general conclusion that in the case of application of mag-MIPs, the most environmentally burdensome stage (the largest amount of consumed solvents and reagents) is the stage of developing a ready-to-use sorption material with magnetic properties. The remaining steps (application and final determination) are more in line with the aspects of GAC by reducing the number of sample preparation steps, reagents consumed (especially during the analytical application), and shortening the analysis time with relatively good LOD and recovery values. Table 3 summarizes the basic information on selected analytical procedures described in the literature related to the use of various types of mag-MIPs for selective binding/collecting and determination of specific types of pesticides in environmental and food samples.

**Figure 6:**
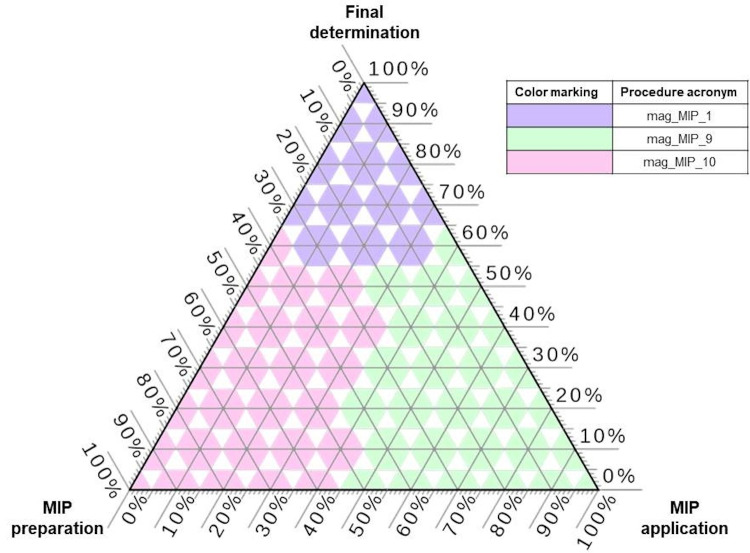
The winning procedures based on mag-MIPs according to TOPSIS rankings for different weights combinations.

Detailed data was collected from the references listed in Table 3 and a database was developed to show those stages of the use of mag-MIPs that have the most significant effect on increasing/decreasing the green character of the entire analytical procedure. Figure 6 represents the winning procedures based on magnetic MIPs for different combinations of TOPSIS weights. The most beneficial procedure according to MIP synthesis criteria is mag_MIP_10 that applied acrylamide as functional monomer that is assessed as greener than methacrylic acid or 2-vinylpyridine. The amounts of functional monomer, crosslinking agent and porogenic agents are low. Considering the sample preparation stage, the winning procedure is mag_MIP_9 that is the only procedure that does not incorporate solvent for elution of analytes. It was caused by the fact that during this stage of sample preparation, deionized water was used as elution medium. For this reason, its negative environmental impact might be omitted. Regarding final determination the most beneficial procedure is mag_MIP_1 that is based on HPLC with fluorescence detection system that allows to obtain reasonable LOD and RSD. The ternary plot is also a hint, which approaches to MIP preparation, sample preparation and final determination are the most beneficial from greenness and metrological perspective and should be preferentially considered during development of analytical procedures.

### 3.3. Quantum and Carbon Dots as a New “Greenest” Solution in the Field of Pesticide Detection Process

**Figure 7:**
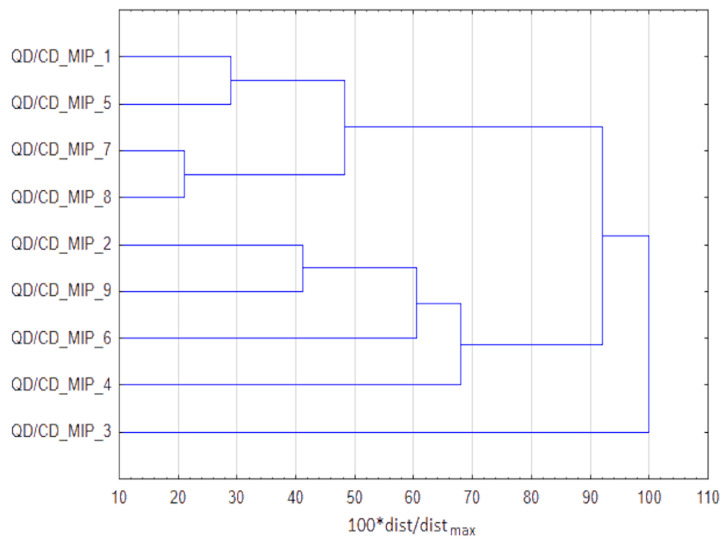
Grouping of procedures based on quantum or carbon dots, obtained with cluster analysis.

The results of grouping with cluster analysis are presented in Figure 7. There are two groups formed, the first one consisting of 4 procedures—1, 5, 7, 8 and the second group consisting of the remaining 4 procedures, keeping in mind that procedure QD/CD_MIP_3 is definitely an outlier. The procedures being in the first group and the outlier are based on reversed microemulsion, while all procedures in the second group are based on sol-gel method. Procedure QD/CD_MIP_3 is an outlier, since it is the only procedure that involves initiator and solvent for the desorption of analytes, what makes it so different (for the discriminators it is needed to see raw dataset gathered based on the information published in papers listed in Table 4).

The application of QDs and/or CDs covered by the suitable imprinted polymer is now one of the newest solutions in the field of almost direct determination of chemical compounds in environmental and food samples. Such materials might be successfully applied as the key elements of optoelectronics as well as fluorescence sensing systems. Xiao et al., 2020 describe in detail the wide spectrum of fluorescent nanomaterials combined with MIP, focusing on their synthesis conditions, analytical applications, and possible challenges such as the complexity synthesis routes [77]. In general, considering the application of QDs or CDs covered with MIP to the analytical practice whole laboratory process might be considered as a green solution. There is no doubt, that their analytical application stage and analytes final determination process are characterized only by several simple steps and consumes small amount of a sample volume. The final determination stage is based on fluorescence measurements performed at the defined excitation wavelength. The most neuralgic is the appropriate preparation of the advanced nanomaterials with an attached imprinted layer. In general, in the case of QDs their structure is made of the core, involving an inorganic material such as CdS, CdSe, CdTe, PbS, PbSe, InP and InAs semiconductors covered by the shell and a cap. The essential optical and semiconductor properties (broad and continuous absorption spectra, narrow and symmetric emission peaks, high photostability) are directly connected to the core characteristic. The core size is also responsible for wavelength of light emitted from QDs. The several solutions for surface modification for QDs might be applied to increase the quality and usability of QDs such as using low molecular ligands or polymer films with thiol groups. The most important limitation of QDs utility is the toxicity of material as a consequence of employment of core heavy atoms (surface oxidation, photodegradation) or hazardous reagents introduced during their preparation process—silanization and introducing the thin film layer of polyethylene glycol (PEG) polymer, stabilization by amphiphilic molecules and polymers, and the ligand exchange method. The solution for this problem is the approach in which the core of the nanomaterial is based on the carbon dots (CDs) due to the low toxicity, good dispersion in water and from the green chemistry point of view the possibility of preparation from natural substances (eco-friendly and biocompatible). The surface of carbon matrix of CDs are mainly functionalized by carboxyl, hydroxyl, amine, ether, carbonyl or polymeric groups to increase their applicability. 

In both cases, QDs and CDs, the most curtail is the surface modification and functionalization. Thanks to this process nanomaterials might reach the appropriate capability to operate in system characterized by the complex composition and be introduced for analytical purposes. In this case, the application of MIPs is considered as a very effective solution—conjugates consist of QD/CD-MIP systems. Generally, in the literature the most commonly used QDs/CDs preparation techniques are classified into two main strategies—top-down approach and bottom-up approach. Detailed information about the characteristics of mentioned preparation strategies is listed elsewhere. Considering the QDs/CDs production strategy based on their low-cost, good efficiency, easily controllable conditions, size and morphology, the bottom-up approach is the most popular solution [78,79]. The final modification (imprinting process) and preparation of core for QD/CD-MIPs synthesis might be performed employing the free radical polymerization, the sol-gel method, and the reverse microemulsion method [63,80,81]. For this reason, considering the application of QDs/CDs MIPs in the analytical practice, the most crucial element is the appropriate preparation and functionalization of desired sorption nanomaterials. 

Yang et al. (2019) developed fluorometric microplate-based dimethoate assay using CdSe/ZnS QDs coated with a suitable MIP material. The appropriate MIP-coated QDs were prepared for further functionalization and surface modification based on the modified via one-pot reversed microemulsion technique. In this preparation technique the continuous phase (7.5 mL cyclohexane cyclohexane), surfactant (1.77 mL Triton X-100, Merck KGaA, Darmstadt, Germany) as well as co-surfactant (0.4 n-hexanol, Merck KGaA, Darmstadt, Germany) were mixed in glass flask. Next, the 200 µL of solution containing 3 mg∙mL^−1^ of CdSe/ZnS QDs was introduced and next 70 μL of TEOS and 100 μL ammonia were put into the glass flask. After stirring, 0.1 mmol of template (dimethoate) was dispersed in 0.2 mL of n-hexanol as well as in 30 μL of functional monomer (APTES). The whole mixture was sealed and stirred for 12 h. After this, created microemulsion reaction system was cracked by introducing 10 mL of acetone. At the end, the template molecules were removed using EtOH twice and extracted with EtOH/acetic acid (9:1, *v*/*v*) [49].

A different, more environmentally friendly solution in the field of nanomaterials was proposed by Shirani et al., 2019. The authors described a novel approach in the field of optical sensor based on silane-doped carbon dots (Si-CDs) covered with suitable imprinted polymer for selective detection of acetamiprid in environmental and agricultural samples [48]. At the beginning, the raw nanomaterials Si-CDs were synthetized in stainless-steel autoclave (heated at 180 °C for 4 h) using 500 μL of APTES combined with 1.0 g of citric acid and 15 mL of deionized water. After the reaction was ended, prepared Si-CDs nanomaterials were storage at 4 °C. Afterward, reverse microemulsion technique was introduced to create the acetamiprid imprint on the surface of freshly prepared nanospheres of Si-CDs. At the beginning, 1.8 mL of Triton X-100 and 7.5 mL of cyclohexane were mixed in reaction vessel. Next, 400 μL of freshly prepared nanomaterials and 50 μL of TEOS with 100 μL ammonium hydroxide were added to the previously mentioned mixture and stirred for 2 h. At the end of the Si-CD-MIPs polymerization mixture, 20 μL of APTES and 5 mg of acetamiprid were introduced. Whole reaction vessel was tightly closed at room temperature and closed overnight. Finally, the microemulsion was cracked with 20 mL acetone. Template molecules were washed out with the use of solvent mixture EtOH:ACN (8:2, *v*:*v*) [47]. 

**Figure 8:**
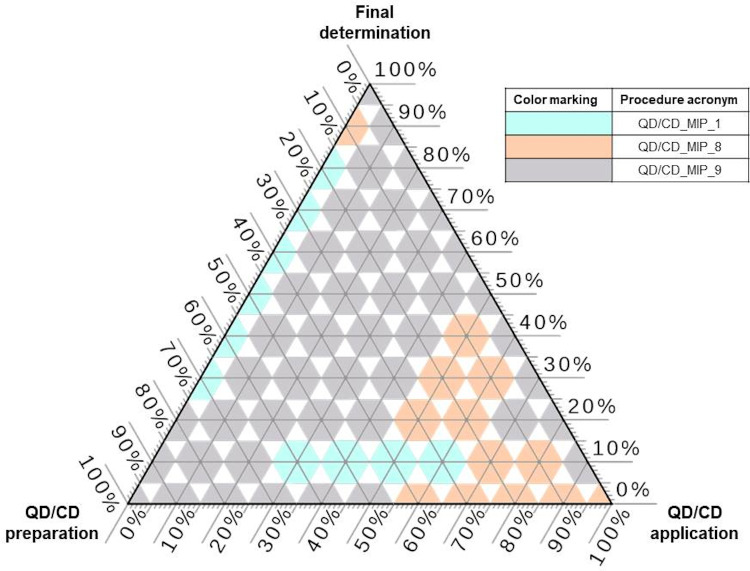
The winning procedures based on quantum or carbon dots application, according to TOPSIS rankings for different weights combinations.

The cited examples of the QD/CD-MIPs study shows the scale of the solvents used (from micro to milliliters) and the advanced degree of preparation of the raw sorption nanomaterial. In most cases, the scale of reagents and solvents consumed during the QD/CD-MIPs synthesis is similar, while the techniques for preparing raw cores may be different. Table 3 provides information on the use in analytical practice of QD/CD-MIPs systems for the determination of selected pesticides in environmental and food samples. Detailed data contained in the papers compiled in Table 4 served as input data for multi-criteria analysis. Figure 8 shows the results of rankings across different sets of criteria. Procedure QD/CD_MIP_9 is the winner for MIP preparation criteria and final determination criteria. Regarding the preparation if MIP, its procedure does not involve any organic solvent but water as porogenic agent, while other procedures use ethanol, methanol or cyclohexane. This procedure has second best RSD and third LOD. QD/CD_MIP_8 is the best alternative if MIP application step is considered. This procedure applied only 10 mg∙L^−1^ of sorbent and just one mL of sample. The ternary plot practically shows that that procedure QD/CD_MIP_9 is the most beneficial from the perspectives of all three stages.

## 4. Conclusions

MIP sorption materials are successfully applied in analytical extractions of pesticides from samples of different matrices, especially from environmental and agricultural samples. CA successfully describes similarities between procedures based on SPE, QDs or CDs and magnetic MIPs and discriminating factors are identified. TOPSIS allows identifying the most beneficial approaches to MIP synthesis, application and final determination, which is a strong hint during development of new procedures based on MIPs. Furthermore, in the case of MIPs form of application MCDA and chemometric techniques gives a possibility to consider the green aspect of the entire MIP material application process. 

From the laboratory practice point of view, it is confirmed that MIP sorption materials introduced in the sample preparation process are regarded as “green” materials. Mainly, due to the fact that they are not single-used tools and the generating of solid wastes is reduced. However, performed TOPSIS analyses and mentioned examples give a reason to consider the MIP not only as a ready-to-use material. It was shown that the form in which the MIP is used, significantly affects their overall “green” character. Based on the obtained database and the results of the statistical and decision-making analysis, additional efforts should be made to ensure that the process of preparation (synthesis) and deposition of imprinted sorbents on carriers (cores) is also considered in terms of the environmental impact of the reagents and reagents used. In the future, it should also be taken into account that the process of obtaining the appropriate imprinted sorption material may fail due to the poorly selected reaction environment (cross-linking agent and porogen/solvent) and inappropriate interactions between the functional monomer and the template molecule. In this case, more advanced calculations and simulations based on computer molecular modelling might be considered as a one of the solution. Nevertheless, as a further stage of work and implementation of the MCDA technique in the process of developing MIP-type sorbents, its usefulness should be checked as an alternative to time-consuming and complex molecular modelling at the stage of searching for an ideal solution, e.g., in terms of structural analogues for difficult to obtain or expensive template molecules.

## Figures and Tables

**Table 1:** Criteria describing for analytical protocols for pesticides determination in environmental and food samples with application of MI-SPE, magnetic MIP and quantum and carbon dots techniques.

Criteria Category	Criterion	Unit	Evaluation System
MIP preparation	Functional monomer type	[points]	NFPA 704
	Functional monomer total amount	[mg]	[-]
	Cross-linking agent type	[points]	NFPA 704
	Cross-linking agent amount	[mg]	[-]
	Porogen agent/solvent type	[points]	NFPA 704
	Porogen agent/solvent amount	[g]	[-]
	Initiator type	[points]	NFPA 704
	Initiator amount	[mg]	[-]
	Bulky polymerization	[points]	binary scale
	Fe_3_O_4_ magnetic microspheres synthesis	[points]	binary scale
	Total amount of solvents and reagents used during surface modification stage	[mL]	[-]
	Removing/washing the unreacted chemicals by organic solvent	[points]	binary scale
	Solid-core amount	[mg]	[-]
	Use of dummy template	[points]	binary scale
	Core type	[points]	0—carbon dots 1—quantum dots
	Quantum dots/carbon dots amount	[µL]	[-]
	Use of surfactant	[points]	binary scale
	The use of breaking microemulsion solvent	[points]	binary scale
	CD/QD@MIP reaction temperature above 40 °C	[points]	binary scale
	Application of semiconductor heavy metals (such as CdTe, CdSe)	[points]	binary scale
MIP application	Sample amount	[mL]	[-]
	Amount of used MIP	[mg]	[-]
	Elution solvent amount	[mL]	[-]
	Additional solvents amount	[mL]	[-]
	Solvent evaporation and/or reconstitution	[points]	binary scale
	Concentration of QD/CD@MIP used	[mg·L^−1^]	[-]
Final determination	Final determination technique	[points]	higher scores assigned to less standard and more environmentally problematic techniques
	Detector	[points]	higher scores assigned to less available detectors
	Amount of injected sample	[µL]	[-]
	LOD value	[µg·kg^−1^]	[-]
	Average RSD value	[%]	[-]
	Recovery in the range from 70–120%	[points]	binary scale

**Table 2:** Selected examples of application of MI-SPE technique in analytical procedures for the determination of pesticide representatives in environmental and agricultural samples.

Procedure Acronym	Sample Type	Analyte	Functional Monomer	Cross-Linking Agent	Porogen/Solvent	Reaction Initiator	Recovery	LOD	Final Determination Technique	References
MI_SPE_1	soil, plant material	imidacloprid	MAA	EDGMA	ACN	AIBN	102–114%	0.03 μg∙g^−1^	IMS	[22]
MI_SPE_2	tap water, soil, cabbage	malathion	MAA	EDGMA	ACN and chloroform	AIBN	96.06–111.49% (tap water) 98.13–103.83% (soil) 84.94–93.69% (cabbage)	0.001 mg∙L^−1^ (tap water), 0.004 mg∙kg^−1^ (soil and cabbage)	GC-FPD	[23]
MI_SPE_3	apple	five benzoylureas pesticides	MAA	EDGMA	ACN	AIBN	69.6–85.9%	0,01 mg∙L^−1^	HPLC-UV	[1]
MI_SPE_4	olive oil	deltamethrin	AA	EDGMA	DCM	AIBN	87–94%	0.95 mg∙L^−1^	HPLC-UV	[24]
MI_SPE_5	almond oil	methidathion, malathion, diazinon	MAA	EDGMA	DCM	AIBN	73–99%	0.3 μg∙kg^−1^	LC-MS/MS	[25]
MI_SPE_6	tap water, river water, municipal wastewater	chlorpyrifos, diazinon, oxon derivatives	MAA	EDGMA	DCM	AIBN	79–104%	0.07 μg∙L^−1^	HPLC-UV	[26]
MI_SPE_7	honey	ethoprophos, phorate, terbufos, dimethoate, malathion, fenamiphos	MAA and Glycidyl methacrylate	EDGMA	chloroform	AIBN	89.2–97.8%	0.0005–0.0019 μg∙mL^−1^	GC-FPD	[27]
MI_SPE_8	grape, green apple	diazinon, quinalphos, chlorpyrifos	MAA	EDGMA	ACN	AIBN	91.51–101.04%	0.83 μg∙L^−1^	HPLC-UV	[28]
MI_SPE_9	lettuce, cucumber	trichlorfon, dichlorvos, dimethoate, imidacloprid, methamidophos	MAA	EDGMA	chloroform	AIBN	87.48–97.85%	0.15 mg∙L^−1^	GC-FPD	[29]
MI_SPE_10	lake water	atrazine	MAA	EDGMA	chloroform	AIBN	90.1–97.8%; 94.4–101.9%	n/m	HPLC-UV	[30]
MI_SPE_11	olive oil	dimethoate, omethoate	IA	EDGMA	DMF	AIBN	89.8–98.02%	0.012 μg∙g^−1^	HPLC-UV	[31]

AA—acrylamide; ACN—acetonitrile; AIBN—2, 2′-azobisisobutyronitrile; DCM—dichloromethane; DMF—dimethylformamide; EDGMA—ethylene glycol dimethacrylate; GC-FPD—Gas chromatography-Flame Photometric Detector; HPLC-UV—high-performance liquid chromatography with ultraviolet detection; IA—itaconic acid; IMS—ion mobility spectrometry; LC-MS/MS—liquid chromatography coupled with Triple Stage Quadrupole Mass Spectrometer; LOD—limit of detection; MAA—methacrylic acid.

**Table 3:** Selected examples of application of magnetic MIPs (mag-MIPs) sorption materials in analytical procedures for the selective recognition of pesticide representatives in environmental and agricultural samples.

Procedure Acronym	Sample Type	Analyte	Functional Monomer	Cross-Linking Agent	Porogen/Solvent	Reaction Initiator	Recovery	LOD	Final Determination Technique	Reference
mag_MIP_1	edible oil	capsaicin, dihydrocapsaicin, eugenol	MAA and AA	EDGMA	toluene	AIBN	87.9–104.1%	0.05685–0.1388 μg∙kg^−1^	HPLC-Fluorescence detector	[32]
mag_MIP_2	orange peel	thiabendazole, carbendazim	MAA	EDGMA	toluene: ACN	AIBN	35%	0.10 mg∙kg^−1^	HPLC-UV	[33]
mag_MIP_3	cucumber	triadimefon, tebuconazole, bitertanol, diniconazole	MAA	TRIM	ACN	AIBN	79.9–110.3%	0.05 μg∙kg^−1^	HPLC-MS/MS	[34]
mag_MIP_4	light and dark honey	thiamethoxam, thiacloprid	2-VP	EDGMA	N,N-dimethylformamide	ABCVA	96.8–106.5%	0.045 μg∙kg^−1^	UHPLC-MS/MS	[35]
mag_MIP_5	supermarket honey	λ-cyhalothrin	AA	DVB	ACN	AIBN	98–107%	2.3 ng∙mL^−1^	fluorescence spectrophotometer	[36]
mag_MIP_6	red wine	methyl parathion, phoxim	APTES	TEOS	MeOH	none	>90%	n/m	HPLC-UV	[37]
mag_MIP_7	rice	chlorpyrifos	MAA	TRIM	EtOH	K_2_S_2_O_8_	81.2–92.1%	0.0072 μg∙g^−1^	HPLC-UV	[38]
mag_MIP_8	soil	methyl parathion	MAA and 4-VP	EDGMA	chloroform	AIBN	81.1–87.0%	5.2 ng∙g^−1^	HPLC-UV	[39]
mag_MIP_9	lake water tap water	2,4-dichlorophenoxyacetic acid	AA	EDGMA	ACN	AIBN	n/m	n/m	HPLC-UVs	[40]
mag_MIP_10	dry red wine	resveratrol	AA	EDGMA	ACN	AIBN	79.3–90.6%	4.42 ng∙mL^−1^	HPLC-UV	[41]
mag_MIP_11	vegetables	acephate	MAA	EDGMA	EtOH	AIBN	89.2–93.4%	0.0025 mg∙kg^−1^	HPLC-UV	[42]
mag_MIP_12	tomato; capsicum; strawberry	ametryn	2-VP	EDGMA	EtOH	AIBN	96–108%	25 nmol∙L^−1^	HPLC-UV	[43]

2-VP—2-vinylpyridine; 4-VP—4-vinylpyridine; AA—acrylamide; ABCVA—4,4′—azobis (4-cyanovaleric acid); ACN—acetonitrile; AIBN—2, 2′-azobisisobutyronitrile; APTES—3-Aminopropyltriethoxysilane; DVB—divinylbenzene; EDGMA—ethylene glycol dimethacrylate; EtOH—ethanol; HPLC-UV—high performance liquid chromatography coupled with ultraviolet detection; HPLC—MS/MS—high performance liquid chromatography coupled with triple quadrupole mass spectrometer; LOD—limit of detection; MAA—methacrylic acid; MeOH—methanol; TEOS—tetraethoxysilane; TRIM—trimethylolpropane trimethacrylate; UHPLC-MS/MS—ultra-high-performance liquid chromatography coupled with tandem mass spectrometry triple-quadrupole.

**Table 4:** Selected examples of introduction of nanomaterial-based MIPs (quantum and carbon dots) in analytical protocols for the specific recognition of pesticide residues in environmental and agricultural samples.

Procedure Acronym	Sample Type	Analyte	Core Type	MIP-QD/CD Preparation Technique	Functional Monomer	Cross-Linking Agent	Porogen/Solvent	Recovery	LOD	Final Determination Technique	Reference
QD/CD_MIP_1	sea water; water well; river water; drinking water	acetamiprid	CQD	reverse microemulsion	APTES	TEOS	cyclohexane	92–102%	0.11 nmol∙L^−1^	Fluoroscence Spectrofotometer	[44]
QD/CD_MIP_2	water and wastewater samples	diniconazole	CdTe/CdS-QDs	sol-gel based method	APTES	TEOS	EtOH and MeOH	95.6–105.5%	6.4 μg∙L^−1^	Fluoroscence Spectrofotometer	[45]
QD/QC_MIP_3	marine sediment	cyfluthrin	FeSe-QDs	reverse microemulsion	APTES and MAA	TEOS and EDGMA	cyclohexane	88.0–113.9%	1.3 μg∙kg^−1^	Fluoroscence Spectrofotometer	[46]
QD/CD_MIP_4	water samples	nicosulfuron	Mn-doped ZnS-QD	sol-gel based method	APTES	TEOS	EtOH	89.6–96.5%	1.1 nmol∙L^−1^	Fluoroscence Spectrofotometer	[47]
QD/QC_MIP_5	wastewater	acetamiprid	Si-CDs	reverse microemulsion	APTES	TEOS	cyclohexane	89.4–101.5%	2 nM	Fluoroscence Spectrofotometer	[48]
QD/CD_MIP_6	apple juice	patulin	Mn-doped ZnS-QD	sol-gel based method	APTES	TEOS	EtOH	102.9–127.2%	0.32 umol∙L^−1^	Fluoroscence Spectrofotometer	[49]
QD/CD_MIP_7	tap water; river water	dimethoate	CdSe/ZnS-QD	reversed phase microemulsion	APTES	TEOS	cyclohexane	89.8–98.0%	2.1 μg∙L^−1^	Fluoroscence Spectrofotometer	[50]
QD/CD_MIP_8	surface river water	pyrethroids	CdSe-QD	reversed microemulsion	APTES	TEOS	cyclohexane	96–102%	3.6 μg∙L^−1^	Fluoroscence Spectrofotometer	[51]
QD/CD_MIP_9	tap water	paraquat	SiO_2_-CdTe-QDs	sol–gel copolymerization process	APTES	TEOS	water	96.4–102.1%	1.94 × 10^−9^ mol∙L^−1^	Fluoroscence Spectrofotometer	[52]

AIBN—2,2′-Azobis(2-methylpropionitrile); APTES—3-aminopropyl-triethoxy-silane; CD—carbon dots; CQD—carbon quantum dots; EtOH—ethanol; MAA—methacrylic acid; MeOH—methanol; TEOS—tetraethoxysilane; QD—Quantum Dots.

## Data Availability

Not applicable.
